# Identification and Assessment of the Potential Allergenicity of 7S Vicilins in Olive (*Olea europaea* L.) Seeds

**DOI:** 10.1155/2016/4946872

**Published:** 2016-02-29

**Authors:** Jose C. Jimenez-Lopez, Adoración Zafra, Lucía Palanco, José Fernando Florido, Juan de Dios Alché

**Affiliations:** ^1^Plant Reproductive Biology Laboratory, Department of Biochemistry, Cell and Molecular Biology of Plants, Estación Experimental del Zaidín, CSIC, 18008 Granada, Spain; ^2^Elayo Group, Castillo de Locubín, 23670 Jaén, Spain; ^3^Allergy Service, Hospital Universitario San Cecilio, 18012 Granada, Spain

## Abstract

Olive seeds, which are a raw material of interest, have been reported to contain 11S seed storage proteins (SSPs). However, the presence of SSPs such as 7S vicilins has not been studied. In this study, following a search in the olive seed transcriptome, 58 sequences corresponding to 7S vicilins were retrieved. A partial sequence was amplified by PCR from olive seed cDNA and subjected to phylogenetic analysis with other sequences. Structural analysis showed that olive 7S vicilin contains 9 *α*-helixes and 22 *β*-sheets. Additionally, 3D structural analysis displayed good superimposition with vicilin models generated from* Pistacia* and* Sesamum*. In order to assess potential allergenicity, T and B epitopes present in these proteins were identified by bioinformatic approaches. Different motifs were observed among the species, as well as some species-specific motifs. Finally, expression analysis of vicilins was carried out in protein extracts obtained from seeds of different species, including the olive. Noticeable bands were observed for all species in the 15–75 kDa MW interval, which were compatible with vicilins. The reactivity of the extracts to sera from patients allergic to nuts was also analysed. The findings with regard to the potential use of olive seed as food are discussed.

## 1. Introduction

The olive tree is a vital element in the environment and agriculture of much of the Mediterranean basin, particularly in Spain. In addition to the high production levels of olive fruit and the quality of its oil [1], extra-virgin olive oil (EVOO) is reputed to have a number of health benefits [2–4].

The specialised olive oil industry is mainly based on crushing olive drupes, which is generally followed by further treatment of the raw material to extract the lipid fraction. However, an increasing body of evidence has highlighted the potential of olive stones as a complementary source of biomolecules with multiple uses and also a role in the biogenesis of EVOO [5, 6]. This has led to the generation of alternative processing techniques such as destoning processes prior to olive milling.

The mature olive fruit, or drupe, is composed of an epidermis surrounding a fleshy mesocarp, in addition to a woody endocarp containing an embryo enclosed by an endosperm with two half-sector hemispheres [7]. The tissues encompassing the olive embryo and endosperm contain a large amount of storage lipids and proteins in the form of compartmentalised structures surrounded by membrane, named oil bodies and protein bodies, respectively [8].

Seed storage proteins (SSPs) in the Poaceae family of monocots and in the dicots plant such as legumes have been widely studied [9]. SSPs have been classified with regard to their molecular masses expressed in terms of their sedimentation coefficients (S) [10] and classically on the basis of their solubility in different solutions. Thus, albumins are soluble in water, globulins in diluted saline, prolamins in alcohol, and glutelins in diluted acid or basis [11]. Previous studies by our research group have demonstrated that 11S SSPs similar to legumins are extremely abundant in the olive endosperm and embryo and are localized in numerous protein bodies present in the cytoplasm of cells, which are part of these tissues [8, 12]. In the olive seed, 11S proteins have been biochemically characterised, and the evolution of their olive seed formation and germination has been determined [8, 13, 14].

SSPs are among the principal food allergens [15, 16], the most important of which within this family are members of 11S, 7S, and 2S SSPs. Their allergenicity is dependent on thermal processing, salt, and high-pressure treatments [17, 18], and particularly gastrointestinal digestion [19]. Many of these proteins have a high level of identity between plant species, resulting in a high level of cross-reactivity [20].

Although more information on olive 11S SSPs has begun to emerge, no up to date data exists about the presence of 7S vicilins in olive. This type of SSPs, widely known as vicilins, can vary in their proportion to 11S SSPs [21]. Neither of these groups is characterised by clear sequence similarity, although they have similar holoprotein structures and a common evolutionary origin [22]. They are both members of the cupin superfamily and have evolved from bacterial enzymes [23]. This study aimed to identify signs of the presence of 7S vicilins in olive seeds at both the transcriptomic and biochemical level and also to assess the putative involvement of these proteins in triggering potential allergenic reactions. This information is considered to be highly useful for investigating the physiology of these seeds and for determining their use as a source of food in the future.

## 2. Materials and Methods

### 2.1. Plant Material

Seeds from 4 species, including* Olea europaea* L.,* Helianthus annuus* L.,* Arachis hypogaea* L., and* Anacardium occidentale*, were used. Olive seeds from the cultivar “Picual” were kindly provided by Elayotecnia S. L. (Castillo de Locubín, Jaén, Spain). Sunflower, peanut, and cashew seeds were purchased from a grocery store.

### 2.2. Retrieval of Sequences from Olive Seed Transcriptome

The search of sequences potentially corresponding to 7S vicilins was carried out in the* de novo* assembled and annotated transcriptome related to olive seeds at different stages of development as well as seed tissues. This transcriptome (unpublished) was generated by the Plant Reproductive Biology group from olive seed cDNA libraries subjected to 454/Roche Titanium+ sequencing reactions. The sequences obtained were preprocessed by using SeqTrimNext to eliminate incorrect, low-quality, and low-complexity readings, linkers, adapters, vector fragments, polyA/polyT tags, and contaminated sequences. The assembly strategy used a combination of different algorithms to correct bias and to produce the best available assembly. To generate primary contigs, MIRA3 (based on overlap-layout-consensus-based algorithms) and de Brujin graph-based Euler-SR software was used. Contigs were further exposed to consensus using CAP3 software. Such strategy was similar to that designed to generate a reproductive transcriptome in olive [24]. Preliminary annotation of the seed transcriptome was used to localise and analyze sequences corresponding to the SSPs of 7S vicilins.

### 2.3. Identity Analysis of Olive Vicilin Sequences

In order to calculate sequence identity, nucleotide and amino acid sequences were aligned by using the ClustalW software program [25–28]. Alignments were carried out among sequences of the same species and between different species. For this purpose, the BLOSUM- (BLOck SUbstitution Matrix-) type substitution/identity matrices [29] were calculated by using the BioEdit software program [30].

### 2.4. Experimental Cloning of Olive 7S Vicilins: Sequencing and Identification of Consensus Sequences

Total RNA was extracted from 0.1 g of olive seeds by following standard methods of extraction and purification using phenol/LiCl [31]. Reverse transcription was performed by using first strand cDNA synthesis (RevertAid Reverse Transcriptase, ThermoScientific) according to the manufacturer's instructions. PCR amplification was carried out in a Mastercycler pro S thermocycler (Eppendorf, Hamburg, Germany) using the following primers designed on the basis of the aligned sequences with the aid of the PRIMER3 program [32]: forward (Oe7S33F) 5′-CAACTTATTTAACAATAG-3′ and reverse (Oe7S11R) 5′-CTAATTGATTGATTATATTC-3′. The PCR products were analysed in 1% TAE-agarose gels, and the unique band of amplification observed was excised, eluted with the MinElute Gel Extraction Kit (Qiagen), cloned into the pGEM-T Easy (Promega) vector, and used to transform competent* E. coli* cells (strain DH5*α*). Blue/white colony selection enabled several colonies harbouring the vicilin sequence to be selected. Plasmid DNA was isolated with the aid of the Real Miniprep Turbo Kit (REAL) and was used for sequencing at the DNA sequencing facilities of the Estación Experimental del Zaidín (CSIC, Granada, Spain). The sequencing readings were again processed with ClustalW in order to generate a consensus sequence, which was used for further bioinformatic analysis.

### 2.5. Phylogenetic Analysis

Olive vicilin consensus sequences were used together with sequences across species to analyze phylogenetic relationships. The amino acid sequence alignment generated by ClustalW was used to generate phylogenetic trees according to the NJ (Neighbour Joining) method [33] based on the BLOSUM-type matrix. Tree visualization was performed using the Treedyn software program [34].

### 2.6. Identification and Selection of Templates for the Generation of 3D Structures

The olive vicilin amino acid sequence was used to search for and select homologous sequences in the PDB (Protein Data Bank) database [35] using the BLAST [36] and SWISS-MODEL [37] servers to identify possible structural patterns. Structural assessment was performed using stereochemical and structural energy parameters [38], and structure comparisons between olive 7S vicilins and 7S vicilin proteins from other plant species were carried out by superimposition of *α* carbon residues in order to calculate the average distances between these C*α* backbones [39].

An initial structural model was generated and checked for error recognition in 3D structures using ProSA (https://prosa.services.came.sbg.ac.at/prosa.php) and also for an initial overall quality estimation of the model using QMEAN (http://swissmodel.expasy.org/qmean/cgi/index.cgi). The final structures of the olive 7S vicilin protein and other proteins were subjected to energy minimization with GROMOS96 force field energy implemented in DeepView/Swiss-PDBViewer v3.7 (http://spdbv.vital-it.ch/) to improve the van der Waals contacts and to correct the stereochemistry of the model. For each sequence analyzed, the quality of the model was assessed by QMEAN, with protein stereology being checked using PROCHECK (http://www.ebi.ac.uk/thornton-srv/software/PROCHECK/) and ProSA (https://prosa.services.came.sbg.ac.at/prosa.php) programs, and protein energy being verified with the aid of ANOLEA (http://protein.bio.puc.cl/cardex/servers/anolea/). The Ramachandran plot statistics for the models were also calculated to show the number of protein residues in the favored regions.

### 2.7. Building 3D Structures Corresponding to 7S Vicilin Proteins

The best structural template (1dquA) was retrieved from the PDB and used to model the 3D structure of the olive seed's 7S vicilin protein by means of the homology modeling approach with the aid of the Workspace application and the SWISS-MODEL automated modeling facility [37]. Visualisation of the 3D model was carried out using the SWISS-PDB Viewer/DeepView program.

### 2.8. Analysis of 2D, Surface, and Electrostatic Potential of Olive 7S Vicilins

The secondary structure was analysed using the Segmer algorithm [40], which compares homologous sequences retrieved from PDB in order to identify conserved substructures. The secondary structure was identified and then compared with the results obtained by the SSpro8 (Scratch Protein Predictor) which adopts the full class output classification of the DSSP [41], PredictProtein [42], NetSurfp [43], and PSIPRED [44] servers.

### 2.9. Identification and Analysis of T- and B-Cell Epitopes

Prediction of regions to the most common alleles of the HLA-DR (human major histocompatibility complex MHC) of an antigenic sequence (first residue of each nonapeptide) was generated by the T-EPITOPE software program [45] based on quantitative matrices and neuronal networks covering a large proportion of the peptides binding to class II human HLA. For the present study, the following most frequent HLA-DR alleles in the caucasian population were selected: DRB1 ^*∗*^0101 (DR1), DRB1 ^*∗*^0301 (DR3), DRB1 ^*∗*^0401 (DR4), DRB1 ^*∗*^0701 (DR7), DRB1 ^*∗*^0801 (DR8), DRB1 ^*∗*^1101 (DR5), and DRB1 ^*∗*^1501 (DR2). The T-EPITOPE algorithm, which predicts nonapeptides binding to these alleles, was used under 5% prediction threshold conditions. In addition, in order to identify the HLA-DR ligands in the 7S vicilin proteins, only peptides binding to at least 3 different haplotypes in the majority of the sequences analyzed were selected [46].

To identify B-cell lineal, or continuous, epitopes, the vicilin sequences were analysed using the Ellipro (http://tools.immuneepitope.org/), ABCpred (http://www.imtech.res.in/raghava/), and COBEpro (http://scratch.proteomics.ics.uci.edu/) programs based on different algorithms [46].

### 2.10. Protein Extraction

The olive seeds were first ground and then defatted by means of supercritical fluid technology (at 300 bar and 55°C) from Elayotecnia S. L. The protein extracts were obtained by stirring 0.1 g of the flour obtained in 1.5 mL of extracting buffer [50 mM phosphate buffer (pH7.8) and 0.2% (p/v) SDS] for 2 h at 4°C. Samples were centrifuged at 1200 ×g for 20 min at 4°C and supernatants were filtered through a 0.22 *μ*m filter (MillexGP, Millipore) and stored at −80°C until use.

Sunflower, peanut, and cashew seeds were first ground and then defatted using hexane. 40 g of ground seeds was mixed with 100 mL of hexane and shaken for 20 min, and the samples were then filtered to remove excess hexane. This step was repeated 3 times. Samples were washed by shaking with distilled water for 20 min and then filtered. This step was repeated a further 3 times. Finally, the flour was washed with 20% ethanol and left to totally dry. Protein extracts were prepared by stirring 0.2 g of flour in 5 mL of extracting buffer [40 mM Tris-HCl (pH7.0), 2% (v/v) Triton-X100, 60 mM DTT, and 10 *μ*L of protease inhibitor cocktail (Sigma)] for 2 hours at 4°C. Samples were centrifuged at 1200 ×g for 20 min at 4°C and stored at −80°C until use.

### 2.11. Protein Quantification

Extracts from olive seeds were quantified using the Bradford method [47]. Alternatively, extracts from sunflower, peanut, and cashew were quantified by using the 2D Quant Kit (Amersham Biosciences) according to the manufacturer's instructions.

### 2.12. SDS-PAGE and Immunoblotting

Proteins were separated by sodium dodecyl sulphate-polyacrylamide gel electrophoresis (SDS-PAGE) using a horizontal ECL system (Amersham) and 4–20% precast gels. 30 *μ*g of total protein was loaded per lane. Gels were stained with Coomassie Blue or transferred to polyvinylidene difluoride (PVDF) membranes. Membranes were blocked with 5% defatted milk in TBST for 1 h at room temperature (RT) and then incubated individually with sera from six different patients. Sera were diluted 1/100 in TBST and 5% defatted milk, and the membranes were incubated overnight at 4°C. Five patients were previously identified as allergic to nuts on the basis of their medical record and complementary assays (ImmunoCAP), with one nonallergic patient being used as the control. Immunodetection was performed using a goat anti-human IgE H&L (HRP) secondary antibody (Abcam) diluted 1/5000 for 1 h at RT. Bands were revealed by using the Clarity Western ECL Substrate (BioRad) and visualized in a ChemiDoc XRS system (BioRad). Images were gathered using a 12-bit CCD camera after 30 minutes of exposure and analyzed with Quantity One software v.4.6.2 (BioRad).

## 3. Results

### 3.1. Identification and Validation of 7S Vicilin Sequences

A* de novo* assembled and annotated olive seed transcriptome (unpublished data) was used to search for sequences allocated to 7S vicilin proteins, vicilins, and vicilin-like proteins. A total of 58 entries were detected, all of which corresponded to partial transcripts (not shown). Further alignment and analysis of the retrieved sequences enabled us to design specific primers, which were used to amplify cDNA from mature olive seeds. PCR resulted in the amplification of a single band of ca. 720 bp. Sequencing of different cloned PCR products facilitated the identification of a partial consensus sequence of 240 amino acids that was used for additional bioinformatic analysis.

### 3.2. Bioinformatic Analysis of the Consensus Olive Vicilin Sequence and Vicilins from Other Species

**Figure 1:**
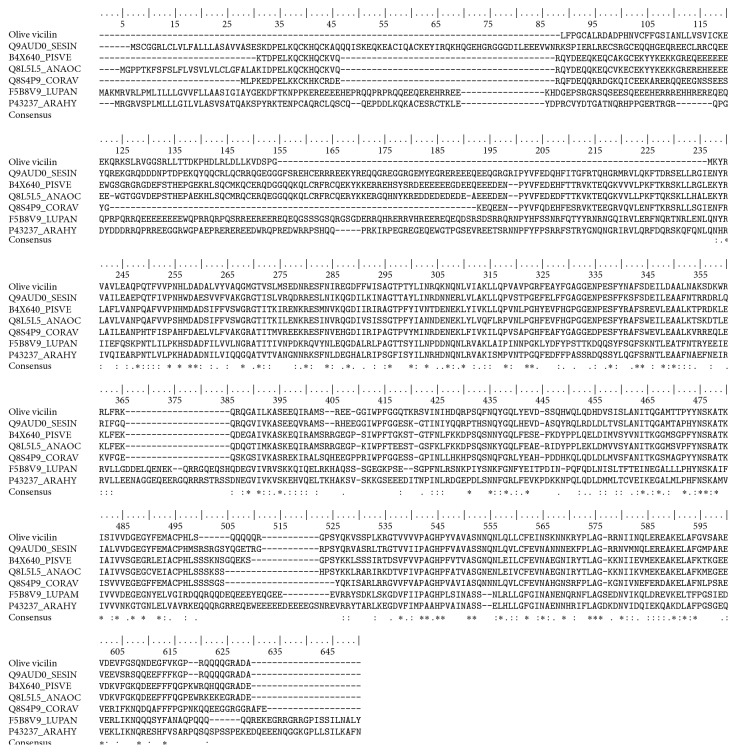
Amino acid sequence alignment of the PCR-validated olive 7S vicilin gene and vicilins from* Sesamum indicum* (SesIn_Q9AUDO),* Pistacia vera* (PisVe_B4X640),* Anacardium occidentale* (AnaOc_Q8L5L5),* Corylus avellana* (CorAv_Q8S4P9),* Lupinus angustifolius* (LupAn_F5B8V9), and* Lupinus albus* (LupAl_Q6EBC1).

The olive 7S vicilin sequence obtained was analysed by using the BLAST (Basic Local Alignment Search Tool) program [48] in order to identify considerable homology with vicilins from other sources. The amino acid sequences selected were used to generate sequence alignments with the aid of the ClustalW program (Figure 1).

**Figure 2:**
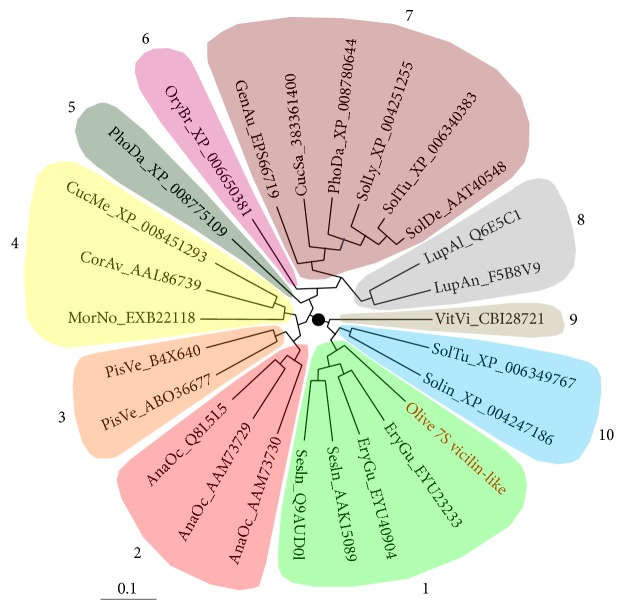
Phylogenetic analysis of 26 amino acid sequences of vicilins from olive and the following plant species: EryGu,* Erythranthe gutta*; SesIn,* Sesamum indicum*; AnaOc,* Anacardium occidentale*; PisVe,* Pistacia vera*; MorNo,* Morus notabilis*; CorAv,* Corylus avellana*; CucMe,* Cucumis melo*; PhoDa,* Phoenix dactylifera*; OryBr,* Oryza brachyantha*; GenAu,* Genlisea aurea*; CucSa,* Cucumis sativa*; SolLy,* Solanum lycopersicum*; SolTu,* Solanum tuberosum*; SolDe,* Solanum demissum*; LupAl,* Lupinus albus*; LupAn,* Lupinus angustifolium*; VitIn,* Vitis vinifera.*

The partial 7S vicilin gene sequence from olive was also used to carry out a phylogenetic analysis aimed at determining its relationship to 7S vicilin proteins from other species (Figure 2). The phylogenetic analysis was performed using the NJ (Neighbour Joining) method [33] based on the BLOSUM-type matrix. A total of 26 amino acid sequences from different plants were used. Ten different phylogenetic groups were identified, with the olive 7S vicilin integrated in Group 1, jointly to four additional sequences from* Erythrante guttata* and* Sesamum indicum*.

A structural assessment to verify the accuracy of the olive 7S vicilin protein model was carried out through a comparative analysis of the template crystallographic structure used to build the model, using stereochemical and energy minimization parameters showing the following data.

Analysis of the best template 1dqu gave a value of 0.636 for the Qmean parameter (linear combination of six terms, including stereology and energy, to estimate model reliability ranging from 0 to 1) and 0.598 for the olive 7S vicilin model. ProSA, another parameter used to check overall structural quality, showed a* Z*-score of −9.06 and −7.95 for the crystallographic structural template and for the olive 7S vicilin structure constructed, respectively. These two parameters show quite similar values when compared to the natural, crystallographic structure and the structure of the olive 7S vicilin model; this means that the olive 7S vicilin model constructed is accurate and close to its template in structural quality terms.

Thus, we also checked the stereochemistry of the model using Procheck analysis, which showed that 80.5, 18.6, 0.7, and 0.2% of template structure residues were located in favorable regions, allowed regions, generally allowed regions, and disallowed regions in the Ramachandran plot, respectively; for the olive 7S vicilin model, these values were 91.1, 7.2, 1.0, and 0.6%, respectively, with even more residues being located in favourable regions and a similar situation for residues in nonfavourable regions.

Taking the comparisons of all these parameters together, it is possible to state that the olive 7S vicilin model built from its crystallographic template is sufficiently accurate to be used in further structural analyses. A similar assessment was made for the other 7S vicilin protein structures built in comparison with their templates.

**Figure 3:**
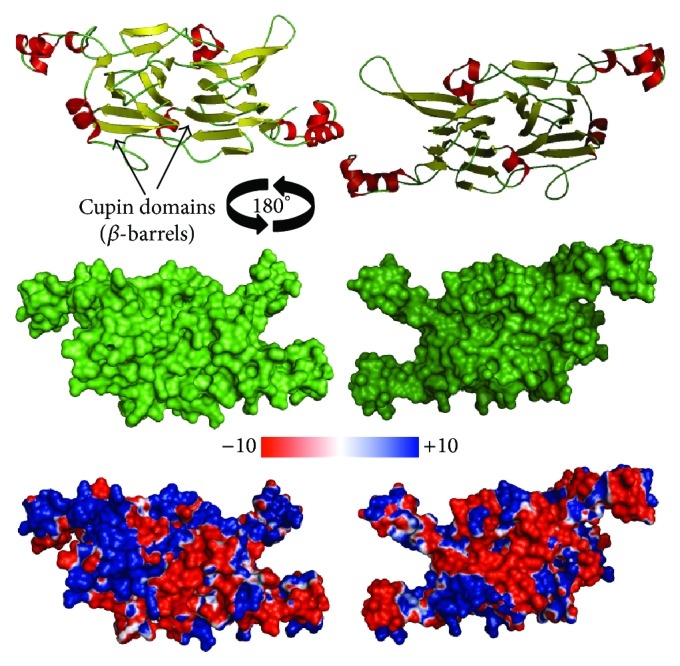
2D structural elements, surface structure, and electrostatic potential analysis of the partial structure of the olive 7S vicilin. Upper panel: 3D structure showing *α*-helixes in red, *β*-sheets in yellow, and coils in green. Middle panel: representation of the protein surface. Lower panel: representation of electrostatic potential. The surface colors represent the charge density, with positive values in blue and negative values in red.

A comparative structural analysis of the partial sequence of the olive vicilin and vicilins from other species was also carried out using homology modeling. In the protein structure of the olive 7S vicilin, a total of 9 *α*-helixes and 22 *β*-sheets were identified (Figure 3, upper panel). We also obtained a representation of the protein fragment surface and its electrostatic potential (Figure 3, middle and lower panels, resp.). Analysis of the latter revealed that prominent charged residues are present in the structure, with over 50% of the side chains exhibiting positive values (Figure 3, lower panel).

Surface electrostatic potential analysis revealed several prominent charged residues, with over half of the side chains showing high positive values in the blue region. Interestingly, a large number of positively charged residues were concentrated on one side of the structure. By assigning a value of +1 to basic residues (Arg, Lys) and −1 to acidic residues (Asp, Glu), the net protein charge was calculated to be +4 for the olive 7S vicilin.

**Figure 4:**
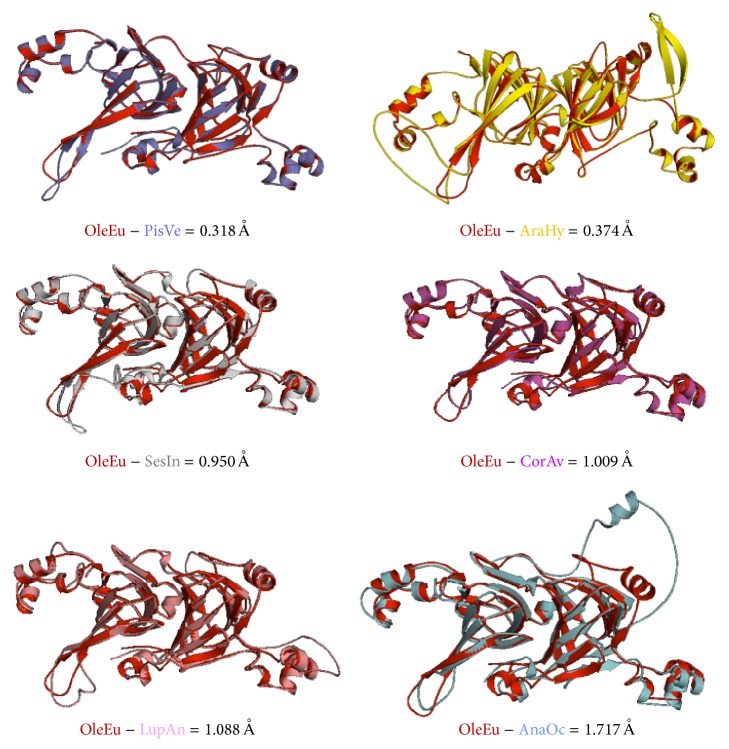
Structural comparison of the olive vicilin fragment with 7S vicilin proteins from other species. Superimpositions of the 3D structure of the olive vicilin (in red) and 3D models generated for the other proteins species are shown.* Pistacia vera* (purple),* Arachis hypogea *(yellow),* Sesamum indicum* (grey),* Corylus avellana* (pink),* Lupinus angustifolius* (light pink), and* Anacardium occidentale* (light blue). Numerical values for the structural differences are also displayed (in Å).

Structural comparison of olive vicilin fragments, through 3D structure superimposition, showed superimposition values ranging from 0.318 Å to 1.717 Å (Figure 4). The best superimposition matches were those corresponding to* Pistacia vera* (0.318 Å) and* Arachis hypogea* (0.374 Å).

All vicilin-like proteins shared a common feature, two b-barrels, each of which is composed of 11 antiparallel *β*-sheets. Both barrels are part of the main globular domain of the protein. This commonly shared feature among vicilin-like proteins is also a characteristic of the whole cupin superfamily. The olive 7S vicilin is a seed storage protein, which, being a nonenzymatic protein, does not exhibit a catalytic domain. Comparison with other vicilin-like proteins showed that most of these 7S vicilin proteins are quite similar in terms of their core structure, or globular domain; major differences between the protein structures compared in this study have been found in the special distribution and the length of some coils/turns and *α*-helixes connecting antiparallel *β*-sheets in the same domain as the 2D structures.

### 3.3. Identification and Analysis of T- and B-Epitopes

Cross-allergenicity and modulation of the allergenic response are highly dependent on the presence of epitopes recognised by the T cells of the human immune system. These epitopes may be present in different orthologs and therefore be responsible for cross-reactivity. In order to determine these putative epitopes and their variability, the method described by Burastero et al. [49] was used with the aid of the T-EPITOPE program [45].

The use of this software program enabled us to identify 19 T-epitopes, 9 of which were shared with more than one of the vicilins analysed (Table 1). Two epitopes (T2 and T9) were ubiquitous and were detected in all vicilins analyzed. The other T-epitopes, which were detected in only one of the vicilins analysed, were therefore considered to be species-specific (Table 2). The olive vicilin fragment showed 7 T-epitopes, 4 of which were found in other species, with 3 T-epitopes being unique to this species.

Analysis of the presence of immunodominant epitope regions recognised by IgG and IgE (B-cell epitopes) in the different vicilins resulted in the identification of 17 epitopes, 11 of which were shared by several 7S vicilin proteins (Table 3). Six other B-cell epitopes, present in particular species, were thus considered to be species-specific (Table 4). The olive vicilin fragment displayed 5 of these epitopes, 2 of which were shared with several other vicilins. Interestingly, 3 of the epitopes were only present in the olive vicilin.

### 3.4. SDS-PAGE Analysis of Protein Extracts from Nuts and Reactivity of Sera from Allergic Patients

**Figure 5:**
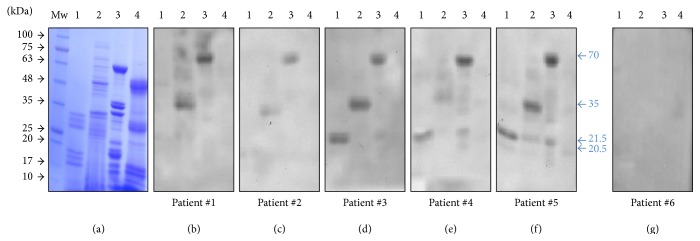
(a) Protein profiles of extracts from different seeds after SDS-PAGE and Coomassie staining. Lane 1: olive seed, Lane 2: sunflower, Lane 3: peanut, and Lane 4: cashew. (b)–(f) Immunoblots containing the same protein extracts as (a), probed with the sera from patients #1, #2, #3, #4, and #5 allergic to nuts. (g) Immunoblot containing the same protein extracts as (a) probed with the serum from a nonallergic patient (negative control).

Figure 5(a) shows the SDS-PAGE profiles of the 4 extracts used in this analysis under denaturing and reducing conditions for the separation of peptides. The 4 profiles are essentially different and contain numerous bands of different intensities. It is important to note the presence of high-intensity bands in the 15–75 kDa range.

The allergograms obtained after probing the immunoblots with the sera of the different patients (Figure 5) are characterized by a wide variability in the reactivity to the extracts. Thus, patient #2 showed lower overall reactivity as compared to the other sera. With regard to the individual extracts, the cashew extract showed low reactivity to any of the patients used to generate the allergograms. On the other hand, sunflower and peanut extracts displayed IgE reactive bands for all the patients tested, with the exception of the nonallergic (control) extract. The olive seed extract contained IgE reactive bands in relation to three of the patients (#3, #4, and #5). The sizes of the highly reactive bands coincided with respect to the olive (ca. 22 kDa), sunflower (ca. 35 kDa), and peanut extracts (ca. 70 kDa), although other minor bands (e.g., 20.5 and 21.5 kDa) were visible in all the allergograms. Immunoblots probed with the sera of a nonallergic patient displayed no reactive bands.

## 4. Discussion

The use of up-to-date transcriptomic data available on olive seeds enabled us to clearly determine that 7S vicilin proteins, or at least vicilin-like proteins, are present in this seed. However, the information available is still scarce: this transcriptome contains only a limited number of transcripts, as it was built on the basis of 454/Roche Titanium+ technology, which was used in order to obtain long readings suitable for building a transcriptome in the absence of genomic data [24]. A larger number of 7S vicilin protein sequences might therefore be present in the olive seeds. In addition, variability among these sequences is likely to be underrepresented in the current data. Next generation sequencing (NGS) developments, currently underway, will provide a better understanding of these proteins in the olive seed and also provide data concerning their variability and expression levels.

To the best of our knowledge, the present study reports for the first time the presence of vicilins in olive seeds. Experimental validation provided us with a partial, though highly significant, sequence of this protein family. The sequence obtained was good enough to make an* in silico* comparative analysis of vicilins from other species. Thus, structural data, genetic relationships, and 3D structural superimposition with the models of other vicilins from different species were obtained. One of the preconditions to obtain 3D PDB models [35] is that, to model the protein, it must share considerable identity (usually over 50%) with templates in the database, a requirement which was widely fulfilled by the olive vicilin sequence obtained. For this reason, the superimpositions carried out can be regarded as reliable. Moreover, the superimposition values are sufficiently low to ensure a good coincidence rate between the molecules tested.

Allergy is a very important issue with regard to vicilin proteins which are regarded as important allergens involved in food allergies [15, 16, 21]. Although olive seed consumption has been highly limited to date, the implementation of new and alternative olive processing methods may lead to this material being used as a food ingredient. It is therefore important to determine the allergenic potential of this material. The* in silico* analysis carried out in this study clearly identifies T- and B-epitopes which are likely to be involved in allergies. Most of the epitopes identified are shared with other vicilins, thus highlighting the similarities between the olive vicilin and other vicilins. Moreover, specific epitopes are also present in this olive protein and other proteins. This finding is consistent with the results obtained following immunoassays of olive seed protein extracts with patients' sera, which may reflect predicted* in silico* heterogeneity. Nevertheless, the differences observed in allergograms cannot be unequivocally attributed to the presence of vicilins, as many other potentially allergenic proteins, such as 11S and 2S SSPs, are present in the assayed seed extracts. Although 7S vicilin proteins range in size from 60 to 75 kDa, other sizes are possible as a result of SDS-PAGE separation carried out under both denaturing and reducing conditions prior to immunoassays. These assays could benefit from using specific antibodies in addition to sera from patients [50, 51] in order to focus on the specificity of the IgE targets. However, to our knowledge, no commercially available antibodies to vicilins are available. Future studies need to address the generation of such antibodies, which would be highly useful molecular tools for diagnostic and research purposes. Other biochemical approaches, including the purification of vicilins, with the aid of chromatographic methods followed by SDS-PAGE and MALDI-TOF/MS identification have not yet been used [52].

## 5. Conclusions

Evidence of the presence of vicilins in olive seeds is supported by the identification of at least 58 sequences in the mature olive seed transcriptome, displaying considerable annotation identity with 7S vicilins from other species. Furthermore, one of these sequences was validated by experimental methods, enabling* in silico* comparative analysis of other vicilins. Although the olive sequence was incomplete, it showed characteristic structural details, a close genetic relationship, and good 3D structural superimposition with the built models of vicilins from different species, suggesting that olive vicilins are, to a great extent, similar to other vicilins. Moreover, both unique and/or commonly shared T- and B-epitopes among other 7S vicilins were present in the olive sequence. These epitopes are likely to be responsible for the reactivity of olive seed extracts to the sera from several patients allergic to nuts despite the fact that olive seeds are not commonly used as a food ingredient.

## Figures and Tables

**Table 1:** T-cell epitopes common to more than one of the vicilins analysed.

Epitope name	Sequence	Shared species
T1	LVIAKLLQP	**Olive_002829** *Corylus avellana*_Q8S4P9 *Arachis hypogea*_P43237
T2	FEMACPHLS	**Olive_002829** *Sesamum indicum*_Q9AUD0 *Corylus avellana*_Q8S4P9
T3	INLLHKHPS	*Corylus avellana*_Q8S4P9 *Lupinus angustifolius*_F5B8V9 *Lupinus albus*_Q6EBC1
T4	YVAVASNNQ	**Olive_002829** *Sesamum indicum*_Q9AUD0
T5	LVIAKLLQP	**Olive_002829** *Sesamum indicum*_Q9AUD0 *Pistacia vera*_B4X640 *Corylus avellana*_Q8S4P9 *Arachis hypogea*_P43237
T6	VVLNGRATI	*Lupinus angustifolius*_F5B8V9 *Lupinus albus*_Q6EBC1, *Arachis hypogea*_P43237
T7	VVLLPKFTQ	*Anacardium occidentale*_Q8L5L5 *Lupinus albus*_Q6EBC1
T8	LRGIENYRV	*Sesamum indicum*_Q9AUD0 *Pistacia vera*_B4X640 *Arachis hypogea*_P43237
T9	LVSVLVLCL	*Anacardium occidentale*_Q8L5L5 *Lupinus albus*_Q6EBC, *Lupinus angustifolius*_F5B8V9

**Table 2:** Species-specific T-cell epitopes in the different species analyzed.

Epitope name	Sequence	Specific species
Ts1	FFGSIANLL	**Olive_002829**
Ts2	YLINRQKNQ	**Olive_002829**
Ts3	WRRLFRKQR	**Olive_002829**
Ts4	IVIVSKKQI	*Lupinus albus*_Q6EBC1
Ts5	MRVRLPMLI	*Lupinus angustifolius*_F5B8V9
Ts6	YVNITKGGM	*Pistacia vera*_B4X640
Ts7	YRLAVLVAN	*Anacardium occidentale*_Q8L5L5
Ts8	VKILQPVSA	*Corylus avellana*_Q8S4P9
Ts9	FVSARP QSQ	*Arachis hypogea*_P43237
Ts10	MVIVVVNKG	*Arachis hypogea*_P43237

**Table 3:** B-cell epitopes identified common to more than one of the vicilins analysed.

Epitope name	Sequence	Shared species
B1	ASESKDPELK	*Sesamum indicum*_Q9AUD0, *Corylus avellana*_Q8S4P9, *Arachis hypogea*_P43237
B2	QQQISKEQKE	*Sesamum indicum*_Q9AUD0, *Pistacia vera*_B4X640, *Anacardium occidentale*_Q8L5L5, *Corylus avellana*_Q8S4P9
B3	RGCEQQHGEQR	*Sesamum indicum*_Q9AUD0, *Pistacia vera*_B4X640, *Anacardium occidentale*_Q8L5L5, *Corylus avellana*_Q8S4P9
B4	RQGEGGGFSR	*Sesamum indicum*_Q9AUD0, *Pistacia vera*_B4X640, *Anacardium occidentale*_Q8L5L5
B5	CERRREEKYREQQGREGGRGEMYEGREREEEQEEQGRGR	*Sesamum indicum*_Q9AUD0, *Pistacia vera*_B4X640, *Anacardium occidentale*_Q8L5L5
B6	GAGGENPESFY	**Olive_002829** *Pistacia vera*_B4X640 *Anacardium occidentale*_Q8L5L5, *Corylus avellana*_Q8S4P9, *Lupinus angustifolius*_F5B8V9
B7	EKQDEGAIVK	*Pistacia vera*_B4X640, *Anacardium occidentale*_Q8L5L5, *Corylus avellana*_Q8S4P9, *Lupinus albus*_Q6EBC1
B8	EDEQEYEEQRRGQEQSDQDE	*Lupinus angustifolius*_F5B8V9, *Lupinus albus*_Q6EBC1, *Arachis hypogea*_P43237
B9	SREEGGIWPFGGQTKR	**Olive_002829** *Corylus avellana*_Q8S4P9 *Lupinus angustifolius*_F5B8V9, *Lupinus albus*_Q6EBC1
B10	DQQRQQDEQEEEYEQGEEEVRR	*Lupinus angustifolius*_F5B8V9, *Lupinus albus*_Q6EBC1, *Arachis hypogea*_P43237
B11	QSYFANGQPQQQQQQQSEKEGRRGRRGSSL	*Lupinus angustifolius*_F5B8V9, *Lupinus albus*_Q6EBC1, *Arachis hypogea*_P43237

**Table 4:** Species-specific B-cell epitopes in the different species analysed.

Epitope name	Sequence	Specific species
Be1	KHQGEHGRGGGDIL	*Sesamum indicum*_Q9AUD0
Be2	DQRPSQFNQ	**Olive_002829**
Be3	QGAMTTPYYNSKA	**Olive_002829**
Be4	EITPDRNPQVQ	*Lupinus albus*_Q6EBC1
Be5	KNNKRYPLA	**Olive_002829**
Be6	CQQEPDDLKQK	*Arachis hypogea*_P43237
